# Twelve quick tips for software design

**DOI:** 10.1371/journal.pcbi.1009809

**Published:** 2022-02-24

**Authors:** Greg Wilson

**Affiliations:** Third Bit, Toronto, Canada; McGill University, CANADA

This is a *PLOS Computational Biology* Software paper.

## Introduction

Most people can lift one kilogram, but would struggle to lift one hundred, and could not lift a thousand without planning and support. Similarly, many researchers who can write a few lines of Python, R, or MATLAB to create a plot struggle to create programs that are a few hundred lines long, and don’t know where to start designing an application made up of dozens or hundreds of files or packages.

The core challenge is that *programming in the large* is qualitatively different from *programming in the small*. As the number of pieces in a program grows, the number of possible interactions between those pieces grows much more quickly because *N* components can be paired in *N*^2^ ways. Programmers who don’t manage this complexity invariably produce software that behaves in unexpected (usually unfortunate) ways and cannot be modified without heroic effort.

This paper presents a dozen tips that can help data scientists design large programs. These tips are taken from published sources like [1,2], the author’s personal experience, and discussions over 35 years with the creators of widely used libraries and applications. They aren’t always appropriate—for example, short scripts written for exploratory data analysis don’t need to worry about unit testing—but if you find yourself sketching data structures on the whiteboard, thinking about how different configuration options interact, or wondering how you’re going to support old releases while working on the new one, these tips may help.

Tip 1: Design after the fact.
Tip 2: Design for people’s cognitive capacity.
Tip 3: Design in coherent levels.
Tip 4: Design for evolution.
Tip 5: Group related information together.
Tip 6: Use common patterns.
Tip 7: Design for delivery.
Tip 8: Design for testability.
Tip 9: Design as if code was data.
Tip 10: Design graphically.
Tip 11: Design with everyone in mind.
Tip 12: Design for contribution.

### Tip 1: Design after the fact

When we are doing research, we often don’t know what the code should do tomorrow until we’ve seen today’s results. It’s therefore often pointless to invest as much in up-front planning for our software as we would in drawing up blueprints for a building. But this isn’t a license to create a tangled mess: We can and should make it *look* as though we had a plan so that the next person who has to read our code will be able to understand it (Box 1) [3].

**Box 1. Challenge and response**

Many designers explain the design of their software by recapitulating its history [7,8]. This is sometimes called *challenge and response*: The only way to understand why something works the way it does is to understand the problems that existed at the time it was written and the tools that were available then. Neither programming languages nor existing graphical notations (Tip 10) are particularly good at capturing this, and while many tools have been written for tracking and managing requirements, none have worked well enough that the average programmer would voluntarily adopt them.

*Refactoring* is the process of reorganizing or rewriting code without changing its externally visible behavior. [4] describes common refactorings, such as “extract function” (i.e., move some code out of the function it is in and put it in a new function so that it can be called separately) and “combine parameters into object” (which we will revisit in Tip 5). Just as the tidying steps in a data pipeline either convert messy data to a tidy layout or move the data from one tidy layout to another [5], most refactoring operations move code toward or between well-defined patterns [6].

### Tip 2: Design for people’s cognitive capacity

Just as computers have hard drives and RAM, human brains have long-term and short-term memory [9]. We only have conscious access to short-term memory, and it is much smaller than most people realize: Early estimates were that the average person could hold 7 ± 2 items in short-term memory at once [10], and more recent estimates put its capacity closer to 4 ± 1.

This limitation leads to the second tip for software design: to ensure that the number of things someone has to remember at any time in order to understand the code fits in short-term memory. For example, if a function takes 37 parameters, then the odds are slim that someone will remember them all and put them in the right order without a lot of trial and error. Breaking the function into pieces, each of which takes only a handful parameters, reduces the *cognitive load*. Similarly, defining default values for parameters (for example, “black” as a default font color) allows people not to worry about them most of the time.

A complementary approach is to create families of functions that all take similar inputs and outputs and then combine them using *pipes*. The pipe operator is one of Unix’s key contributions to computing [11], and the idea has been adopted by many other programming systems, including the “tidyverse” family of packages in R [5]. Where a mathematician would write *f*(*g*(*h*(*x*))), a programmer using pipes would write *h*(*x*)|*g*|*f* so that the functions’ names appear in the order in which they are applied. As a result, the reader only has to hold one piece of information in their head at a time, which frees up more space for worrying about other things.

### Tip 3: Design in coherent levels

Another tip for designing functions is that each function should be short, shallow, and single-purpose, i.e., it should implement a single mental operation so that it only takes up a single slot in short-term memory. The easiest way to check if this tip is being followed is to read the function aloud and ask whether all the steps are at the same conceptual level. For example, if I read this Python function aloud:

def main():

 config = buildConfiguration(sys.argv)

 state = initializeState(config)

 while config.currentTime < config.haltTime:

  updateState(config, state)

 report(config, state)

the highlighted comparison of the current time to the halting time in the while loop feels like it’s at a lower level of detail than the function calls. I would probably replace the comparison with a function called something like stillEvolving:

def main():

 config = buildConfiguration(sys.argv)

 state = initializeState(config)

 while stillEvolving(config, state):

  updateState(config, state)

 report(config, state)

This rewrite saves the reader from having to jump between two different levels of detail while they’re trying to figure out what this function does (Box 2).

**Box 2. Expert designers’ thought processes**

While the final design should be leveled, the process used to create it almost certainly won’t be. Studies of expert designers have found that they constantly drill down from high-level ideas to concrete implications to find out whether their plan will work or not [12].

### Tip 4: Design for evolution

Replacing a low-level test with a function as shown above also makes future evolution easier. If we decide that the simulation should run until a specified time *or* until its state has stabilized, we can make that change in the stillEvolving function without modifying anything else.

Hiding details like this is another general rule of software design. Software changes over time because our problems change, our environments change, and because our skills improve. A good design makes independent evolution of parts easier: Basically, a fix *here* shouldn’t require changes *there*. More realistically, a change in one place should only require a small number of changes in a few predictable places.

Software designers achieve this in two ways. *Information hiding* means putting the data each part of a program needs in one place, and only accessing that data through a small set of functions. Doing this reduces cognitive load by allowing people to focus on one part of the program at a time. *Loose coupling* means making those parts independent of one another so that they can be combined in many different ways. Again, one of the reasons the Unix command line and R’s tidyverse have been so successful is how easily their elements can be rearranged to do different things.

Programmers usually implement these principles by separating *interface* from *implementation*. The interface specifies what a piece of software can do; its implementation is how it achieves that, and no other piece of software should be tied to its implementation details. The goal is to enable the construction of software components that can be mixed and matched in the way that USB interfaces allow pieces of hardware to be combined. Many of the more advanced features of programming languages exist to support or enforce this, such as being able to derive classes from one another in object-oriented languages like Python, or creating generic functions that do the same logical thing in different ways for different types of data in languages like R and Julia.

*Design by contract* is one way to enforce this idea [13]. Any function can be characterized by the *preconditions* that must be true of its inputs in order for it to run and the *postconditions* that it guarantees will be true of its output. For example, a function might require that its input be an array of numbers in sorted order, and its postcondition might be that it returns the median of those numbers. If the input isn’t an array, doesn’t contain numbers, or isn’t sorted, the function may not work; if the input satisfies all three conditions, the output is guaranteed to be the median.

If design by contract is followed, then a new version of this function can only *weaken* the preconditions, i.e., place *fewer* restrictions on what it accepts. This rule ensures that the new function will be able to handle everything that the old one could: If the new version placed extra restrictions, such as requiring all the numbers in the input to be positive as well as sorted, then the new version might reject some inputs that the original version would accept.

Similarly, a new version of the function can only *strengthen* the postconditions, i.e., make *more* guarantees about its outputs so that the values the new version produces are a subset of the values the old version could produce. For example, if the old version of the function returned positive numbers, the new version could guarantee to return positive numbers in the range zero to one, but could *not* return negative numbers as well as positive ones. This rule ensures that anything using the old function’s output will still be able to handle everything the new one produces.

### Tip 5: Group related information together

If several things are closely related or frequently occur together, our brains combine them into a “chunk” that only takes up one slot in short-term memory [14]. We can aid this by combining related values into data structures. For example, instead of storing the X, Y, and Z coordinates of points separately like this:

def enclose (x0, y0, z0, x1, y1, z1, nearness):

…

we can store each point’s coordinates in an object and write our code like this:

def enclose (p0, p1, nearness):

…

This allows us to think about each point as one “thing”. Where we need the individual coordinates, we can refer to them as p0.X, p0.Y, and so on.

Grouping related information together also aids code evolution (Tip 4). If, for example, we decide to use radial coordinates instead of Cartesian coordinates, we can change how points are represented without changing most of the functions that pass them around.

### Tip 6: Use common patterns

Some chunks appear so often that we call them “patterns” and give them names (Box 3). Good programmers use *design patterns* to structure their code, both to reduce the amount of thinking they have to do and because these patterns have proven to be useful in the past. Learning patterns helps make someone a better programmer, i.e., there is causation, not just correlation [15]. Conforming to widely understood patterns also makes code more comprehensible, just as dividing this paper into sections and a bibliography that are consistent with what you’ve read before makes it easier to read.

**Box 3. Patterns and precision**

Patterns don’t have to be precisely defined in order to be useful. A classic example is concept of a porch [16]: Two people might disagree over whether a specific structure is one or not, but the idea is still useful.

Patterns can be found at all scales of programming. For example, introductory programming courses often show a pattern for finding the “most valuable” element of a collection, such as the minimum or maximum [17], while “filter the rows, group them by category, and calculate a summary for each group” is a common pattern in introductory data analysis. In more advanced courses, students may be introduced to object-oriented design patterns for traversing complex data structures or using one set of objects to hide the details of how another set of objects work [18,19].

But design patterns can be a mixed blessing. First, our brains try so hard to match inputs to patterns that they will sometimes misclassify things. For example, it once took me the better part of an hour to spot the error in this code:

for (i = 0; i<a.width; i++) {

 for (j = 0; i<a.height; j++) {

  a[i][j] = cos(abs(a[i][j])—lemaitre(b_norm, a[j][i]))

 }

}

The problem is in the second line, which mistakenly uses the variable i where it should use the variable j (Box 4). I couldn’t find the mistake because the code was so close to what it should have been that my brain “corrected” what my eyes were seeing, and because I assumed that since the third line was the most complicated, the error had to be there.

**Box 4. Naming conventions**

In the same way that you shouldn’t write a math problem with a function x of variable g, you should use the same naming conventions as your peers to make your code easier to read. It doesn’t matter what these are, any more than it matters whether you spell “color” the correct way or the British way; what matters is the predictability that comes from consistency.

This example illustrates a corollary to Tip 2: We should write programs that maximize the ratio of unique code to boilerplate. In most modern languages, the five lines shown above can be written as:

a = cos(abs(a)—lemaitre(b_norm, a.transpose()))

This version is probably as efficient as the first, but it is much easier to read and therefore much less likely to mislead.

### Tip 7: Design for delivery

Developer operations (DevOps) has become a buzzword in the last few years. Like “data science” or “computational thinking”, the term is popular because people can use it to mean whatever they want, but the core idea is a good one: managing compilation, packaging, distribution, deployment, and monitoring to make them faster and more reliable [20,21]. Automating these tasks pays off many times over, but only if you design things so that they can be automated. [22] lays out some rules for doing this, and a few others include:

1. Use the same tools to build packages as everyone else who uses your language, for example, pip for Python or devtools for R.
2. Organize your source files in the way your build system expects so that those tools can do their job.
3. Use a logging library rather than print commands to report what your program is doing and any errors it has encountered. Logging libraries allow you to enable and disable certain categories of messages selectively. This is very useful during development, but even more so in production, since it lets whoever is using your software turn reporting on without having to rebuild anything.

### Tip 8: Design for testability

Research software is notoriously difficult to test [23,24], in part because its developers often don’t know precisely what output the code is supposed to produce. (As a frustrated chemist once said to the author, “If I knew what the right answer was, I’d have published it already.”) It *is* possible to test the “other 90%” of a typical data science application—the parts that parse input files, clean up messy data, and so on, but only if those parts are designed with testing in mind.

*Legacy code* is software that we’re afraid to try to modify because it’s hard to understand and things will break unexpectedly. A comprehensive set of tests makes us less afraid [25], but we can only create those tests if we design the software in testable pieces. We can check how well we’ve done this by asking:

- How easy is it to create a *fixture* (i.e., the input that a test runs on)?
- How easy is it to invoke just the behavior we want?
- How easy is it to check the result?
- How easy is it to figure out what “right” is?
- How easy is it to delete the feature?

For example, suppose that our program uses species data stored in a large database that takes several seconds to query. In order to make our program easier to test, we can create a second version of the lookup function that returns hard-coded values for the two or three species used in testing. When the program is running in production, it uses the original function; when we are testing, we swap out that function and use the testing version instead. This trick only works if *all* requests for species information are made via that function, but doing this ensures that if we want to change how information is looked up in future we can be certain that we’ll only have to modify one small piece of code.

### Tip 9: Design as if code was data

The insight on which all modern computing is based is that code is just another kind of data. Programs are just text files, which means we can process it like any other piece of text. Examples of this include:

- style-checking tools that check that the layout, variable names, and other properties conform to coding standards;
- documentation tools that extract specially formatted comments and create cross-referenced manual pages; and
- indexing and navigation tools that enable us to jump directly to the definition of a function or variable.

Similarly, once a program is loaded into memory, it is just another data structure: Instead of interpreting its bytes as characters or pixels, we interpret them as instructions, but they’re still just bytes. This fact impacts design in many ways:

- Passing functions as arguments to other functions so that common operations only have to be written once.
- Storing functions in data structures so that new operations can be added to a program without changing any of the preexisting code.
- Loading modules based on configuration parameters (as in the earlier example of getting species information).

For example, if we want to count the number of positive values in an array, we can write:

def count_positive(array):

 number = 0

 for value in array:

  if value > = 0:

   number = number + 1

return number

If we want to count the number that are negative, we could write a count_negative function that differed by only one character (replacing the > with <). Alternatively, we could write a generic function like this:

def count_interesting(array, test):

 number = 0

 for value in array:

  if test(value):

   number = number + 1

 return number

then put each test in a function like this:

def is_positive(value):

 return value > = 0

and then write something like:

count_interesting(pressures, is_positive)

Many features in modern languages, such as lazy evaluation in R or decorators in Python, leverage this insight, and taking advantage of it can make code much smaller and easier to understand—but only if you remember that what is powerful in the hands of experts is spooky action-at-a-distance for novices.

**Fig 1:**
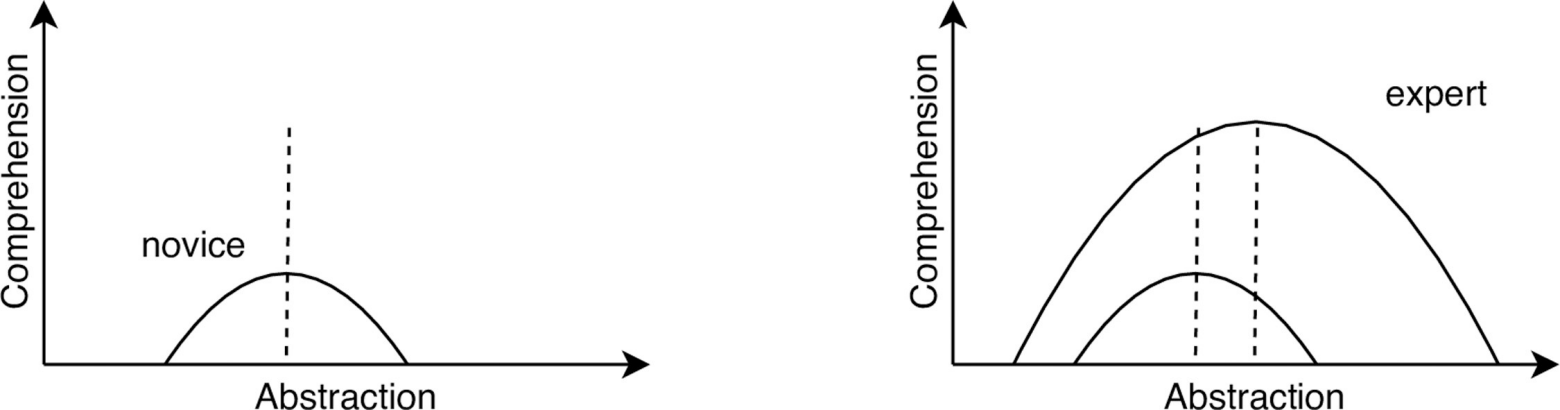
Comprehension curves for novices and experts.

The best balance between abstraction and detail depends on how much people already know. For a novice, too many low-level details obscure the meaning, while too much abstraction makes it impossible to figure out what’s actually going on (Fig 1). As that person gains experience, they become better able to synthesize meaning from detail and translate generalities into specifics, but their optimum balance also shifts. Software that is easiest for them to understand may not be optimal for someone else.

### Tip 10: Design graphically

Many formal graphical notations for software have been designed over the years. The most famous is the Unified Modeling Language (UML), but in practice, it is taught more often than it is used, and when it *is* used, it is usually not in the ways its creators intended [26].

**Fig 2:**
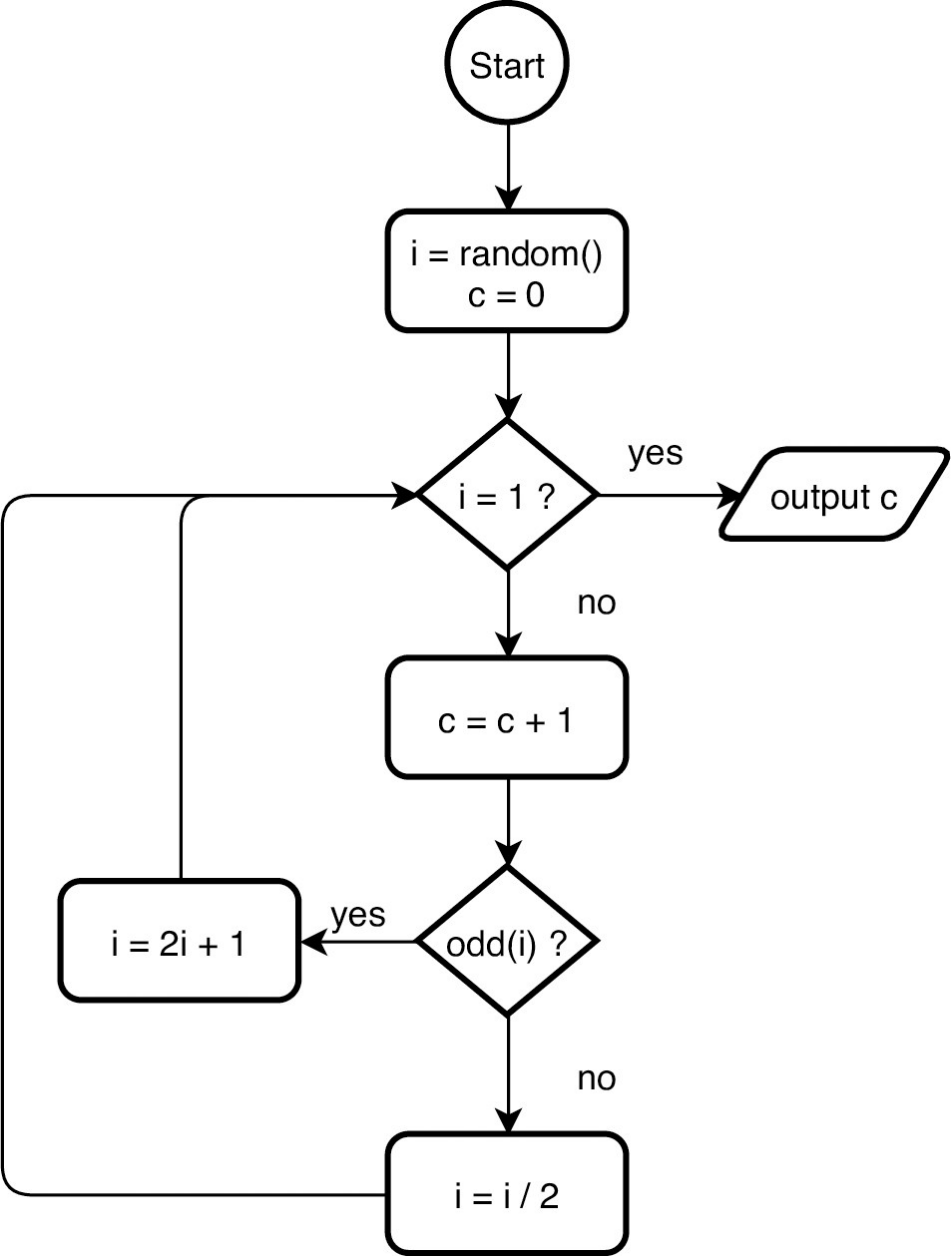
Flowchart.

**Fig 3:**
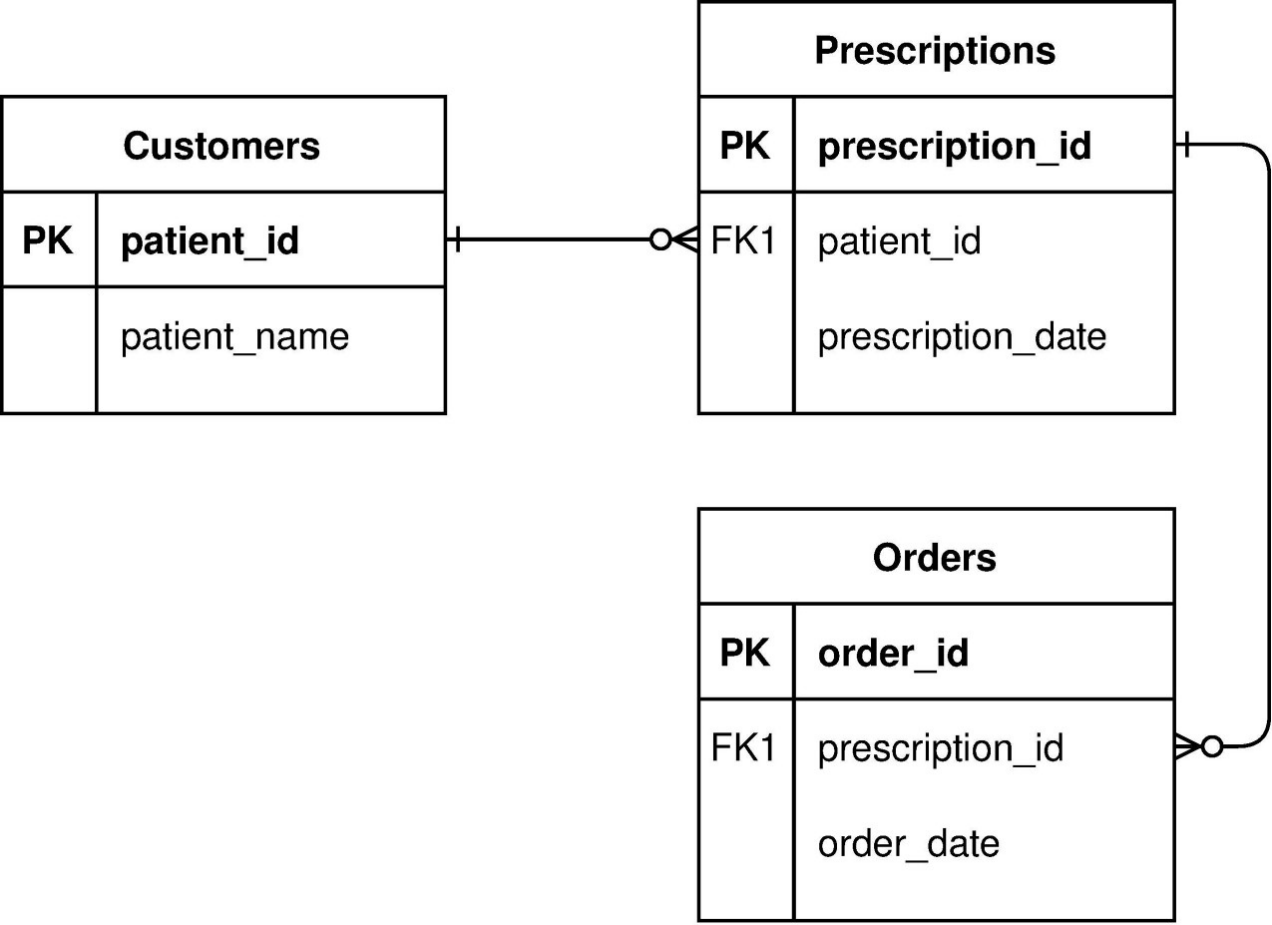
Entity-relationship diagram.

**Fig 4:**
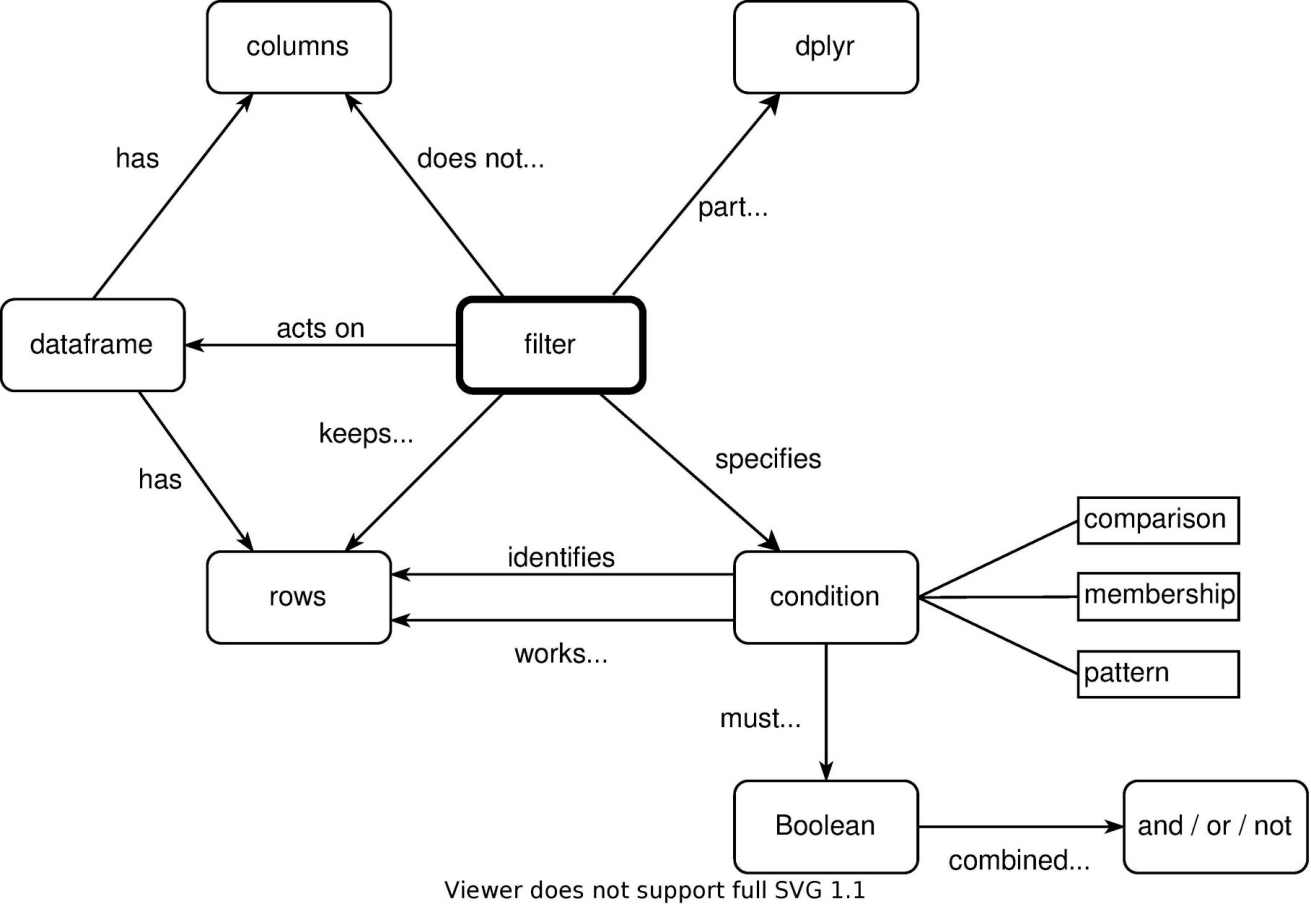
Concept map.

However, many programmers do sketch when they’re designing, and these sketches do help them design. These sketches are usually not meant as blueprints: Instead, they help people *externalize cognition*, i.e., get their thoughts out where they can see them [27,28]. Among the drawings that working programmers often find helpful are:

- flowcharts, which are unfairly maligned [29] (Fig 2);
- entity-relationship diagrams showing how database tables relate to one another (Fig 3);
- concept maps showing how the designer thinks about the overall problem (Fig 4); and
- many others, such as dataflow diagrams, system architecture diagrams showing the major components of an application, and use case maps that show how activity flows through an architecture [30].

### Tip 11: Design with everyone

If the last few years have taught us anything about software, it’s that fairness, privacy, and security cannot be sprinkled on after the fact. For example, if a program is initially designed in a way that allows every user to see everyone else’s data, adding privacy controls later will be expensive and almost certainly buggy. Programmers call this the *Principle of Least Privilege*: Every component in the system should require as few permissions as possible for as short as possible.

But there is much more to safety-conscious design than just data protection. For example, if an application requires users to change their password every few weeks, *security fatigue* will soon set in and people will choose less and less secure passwords [31]. Similarly, programs should not email files to people: Doing that trains them to open attachments, which is a common channel for attacks. And in many jurisdictions, application must allow people to erase data, which means its data structures and database tables have to be designed to allow for actual erasure, not just “mark as inactive”.

Accessibility also can’t be sprinkled onto software after the fact. Close your eyes and try to navigate your institution’s website. Now imagine having to do that all day, every day. Imagine trying to use a computer when your hands are crippled by arthritis. Better yet, don’t imagine it: Have one of your teammates tape some popsicle sticks to your fingers so you can’t bend them and see what it’s like to reply to an email.

Making software accessible doesn’t just help people with obvious disabilities: The population is aging, and everything you do to help people who are deaf also helps people who are gradually losing their hearing [32]. [33] is a good short guide for accessible design: Each poster in this series lays out a few simple do’s and don’ts that will help make your software accessible to people who are neurodivergent, use screen readers, are dyslexic, have physical or motor challenges, or are hard of hearing.

### Tip 12: Design for contribution

Study after study has shown that diversity improves outcomes in fields from business to healthcare because many perspectives make for fewer errors [34,35]. Good design makes it easier for people who aren’t already immersed in your project to figure out where and how they can contribute to it [36], but just as good programmers consider things like packaging and deployment in their designs, so too do they think about the factors that influence contribution:

- Software licensing is a design issue, since a program can’t use libraries whose licenses are incompatible with its own. Many designers therefore prefer permissive licenses like the MIT License to maximize the number of people who will be able to take advantage of their work.
- Applications that support plug-ins—i.e., that allow people to write small modules that the main program can load and use—are often easier for newcomers to contribute to. Similarly, libraries with strong and consistent conventions for passing data (like Unix command-line tools or R’s tidyverse functions) enable people to start with small contributions.

Designing with contribution in mind is also essential to designing inclusively. Many well-meaning attempts at inclusive design fall short because the designers don’t involve the people they intend to help in the process. Nobody can simultaneously be an expert on programming, a research domain, and the needs of multiple overlapping diversities. Asking for help early on maximizes your chance to get things right; designing in ways that make contribution easy maximizes your chances that people who know more than you will share their insights.
